# 108 Development and Implementation of a Burn Nurse Educator

**DOI:** 10.1093/jbcr/irac012.111

**Published:** 2022-03-23

**Authors:** Emily Snyder, Jennifer Rosenthal

**Affiliations:** Medical City Plano, Plano, Texas; Medical City Plano, The colony, Texas

## Abstract

**Introduction:**

Prior to the introduction of the Burn Nurse Educator (BNE), at a growing and newly established burn center, the education of the Burn Trauma ICU staff was completed by the Burn Director and Burn Supervisor. In this burn center, large amounts of education became difficult to create and distribute by the current Burn Director and Burn Supervisor due to the demand of their respective job roles. The role of the BNE was to create initial and ongoing education for the Burn Trauma ICU Staff, Ortho-Trauma staff, ED staff, local EMS agencies, and the community.

**Methods:**

The Burn Nurse Educator reviewed previous education that was provided to the staff, compared it to the Burn Nurse Competencies established by the American Burn Association, and was able to formulate a new education plan. The Burn Nurse Educator created several burn-based courses. These classes included: *Floating into Burn Care*, *Burn Boot Camp*, and *Burns in the Pediatric Population*. There was also the introduction of mock codes focused on the pediatric burn population. In addition to the formulation of these educational opportunities, the Burn Nurse Educator worked directly with the Quality Improvement Committee to find gaps in care. These gaps were then turned into project improvement plans and additional education was provided to the bedside staff. The Burn Nurse Educator formed relationships with local EMS agencies and was able to provide burn lectures and continuing education.

**Results:**

Each class offered had a pre and post test administered, all with improved scores. A sample size of 30 nurses who enrolled in *Burns in the Pediatric Population* had a score increase from 46% to 94%. A sample size of 14 nurses had a score increase of 55% to 80% after enrolling in *Floating into Burn Care*. A sample size of 30 nurses who enrolled in *Burn Boot Camp* had a score increase of 71% to 95%. More importantly, the staff expressed a higher level of confidence when caring for a burn patient after these classes. The outreach with local EMS agencies also increased our EMS admits to the hospital, improved knowledge for caring for burn victims, and created a relationship with our local cities EMS.

**Conclusions:**

It is anticipated that as the program continues to expand, the role of the Burn Nurse Educator will continue to grow and encompass new responsibilities. To ensure each nurse remains competent in their skill set, additional knowledge testing will be completed one year after a nurse has completed a class.

